# The Inter-Continental American Medical Congress

**Published:** 1891-07

**Authors:** 


### THE INTER-CONTINENTAL AMERICAN MEDICAL CONGRESS.

It will be remembered that we referred to this proposed Medical Congress in our editorial account of the meeting of the American Medical Association in the June number. The following circular has been issued:

The Inter-Continental American Medical Congress.

Office of the Chairman of the Committee on Permanent Organization,

Cincinnati, June 6, 1891.

The Committee appointed by the American Medical Association to effect a permanent organization of the Inter-Continental American Medical Congress, met at "The Arlington," Washington, May 7, 1891. The following officers were elected: Charles A. L. Reed, M. D., Cincinnati, O., Chairman; J. W. Carhart, M. D., Lampasas, Texas, Secretary; I. N. Love, M. D., St. Louis, Mo., Treasurer.

On motion, the officers were appointed a special committee to draft a Constitution, and report the same at an adjourned meeting of the general committee, to be held at St. Louis, Mo., Wednesday, October 14, 1891, when the time and place of meeting of the Congress will be decided, and permanent officers elected.

Charles A. L. Reed, M. D., *Chairman.*
J. W. Carhart, M. D., *Secretary.*

At this meeting of the Committee, which is called coincidentally with the meeting of the Mississippi Valley Medical Association, it is expected that definite plans will be discussed looking to the complete organization of the Congress. A full attendance of the Committee is desired.

